# A Novel Technique for the Treatment of a Case of Verneuil's Disease of Perineum and Axillary Regions

**DOI:** 10.1055/s-0041-1731400

**Published:** 2021-07-19

**Authors:** Etienne El-Helou, Alaa Kansoun, Elissa Abi Fadel, Ali Nassif, Houssam Bashir Mazraani, Georges Robert Neaime, Houssein Amin Kassem Moussa, Georges Bassil, Serge Ibrahim, Georges R. Assaf, Houssam Alam

**Affiliations:** 1 Department of General Surgery, Lebanese University, Faculty of Medical Sciences, Beirut, Lebanon; 2 Department of General Medicine, Lebanese University, Faculty of Medical Sciences, Beirut, Lebanon; 3 Department of Anesthesiology, Centre Hospitalier Universitaire Geitaoui, Lebanon; 4 Department of General Surgery, Centre Hospitalier Universitaire Geitaoui, Lebanon

**Keywords:** Verneuil, hidradenitis suppurativa, Etanercept, Integra, synthetic, case report, novel technique

## Abstract

Hidradenitis suppurativa (HS) is a chronic inflammatory disease involving apocrine glands of the skin. It carries out an important burden on the daily life of the patient. Unfortunately, it presents a major concern for medical care management in the absence of clear guidelines for proper medical and surgical treatment. Hence, we report a case of concomitant axillary and perianal HS. We opted for surgical management using a novel technique, which proved efficacy for a year of follow-up recurrence free.

Hidradenitis suppurativa (HS), acne inversa, and Verneuil's disease are relatively the identical terminologies‎. 1 It was described first in the 19th century by the French surgeon Aristide Auguste Stanislas Verneuil‎ 2 and since then numerous guidelines were reported worldwide concerning the management. However, they failed to prove efficacy‎. 1 We report a case of a young male, with wide deep hidradenitis suppurativa of perineum and axillae, managed and followed out for 1 year, without recurrence.

## Case Description

We report a case of a 30-year-old male with a history of hidradenitis suppurativa presented to our clinics with flare up of his disease with perineal and bilateral axillary ulcerative and purulent lesions. The history of his disease dates back to the age of 18 when he was treated medically with Etanercept and kept off symptoms for 10 years, after which he was treated surgically with repetitive incisions and debridement of recurrent small abscesses until this presentation.

On physical examination, the patient was febrile (temperature = 40.1°C), tachycardic (110 beats per minute), normotensive (blood pressure = 120/80 mmHg), and he had normal oxygen saturation (SpO _2_ = 97%). Multiple scares were noted over all his body, large bilateral axillary Hurley stage 2 abscesses, and perineal Hurley stage 3 lesions were noted. They were hot and tender to touch and with purulent secretions.

**Fig. 1:**
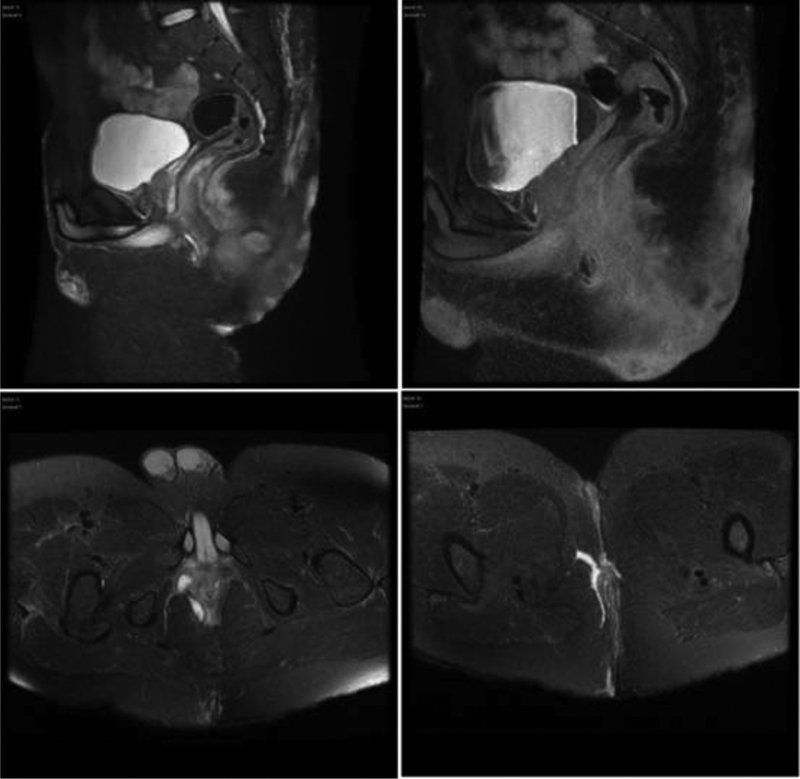
Magnetic resonance imaging of pelvis showing multiple fistulous tract and abscesses.

Moreover, the patient's laboratory studies revealed leukocytosis (white blood cell = 21,200/μL) and CRP (220 mg/L). Interestingly, Magnetic resonance imaging of the pelvis showed complex trans-sphincteric fistula at 12 o'clock with the presence of intersphincteric, anterior perirectal, and right ischial anal fossa abscesses as well as a fistulous tract within the right perineum, right buttocks, and extending from the perineum anteriorly to the intergluteal cleft posteriorly ( Fig. 1 ). Computed tomography of the chest showed cutaneous thickening associated with multiple air pockets bilaterally and almost symmetrically, with evidence of well circumscribed collections.

Broad spectrum antibiotherapy was started as well as multiple attempts of debridement, within few days interval, as a primary treatment of sepsis. Culture samples were in favor of carbapenem-resistant Enterobacteriaceae growth.

**Fig. 2:**
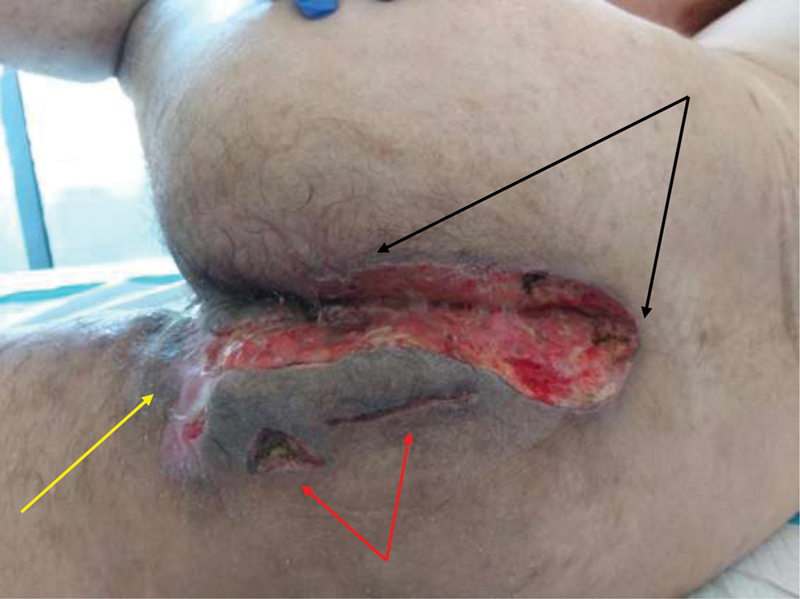
Postdebridement of perineal abscesses (black arrows), after secondary healing of drained perianal lesion (yellow arrow), and multiple incision and drainage sites (red arrows). This photo is 1 day before surgical creation of colostomy.

**Fig. 3:**
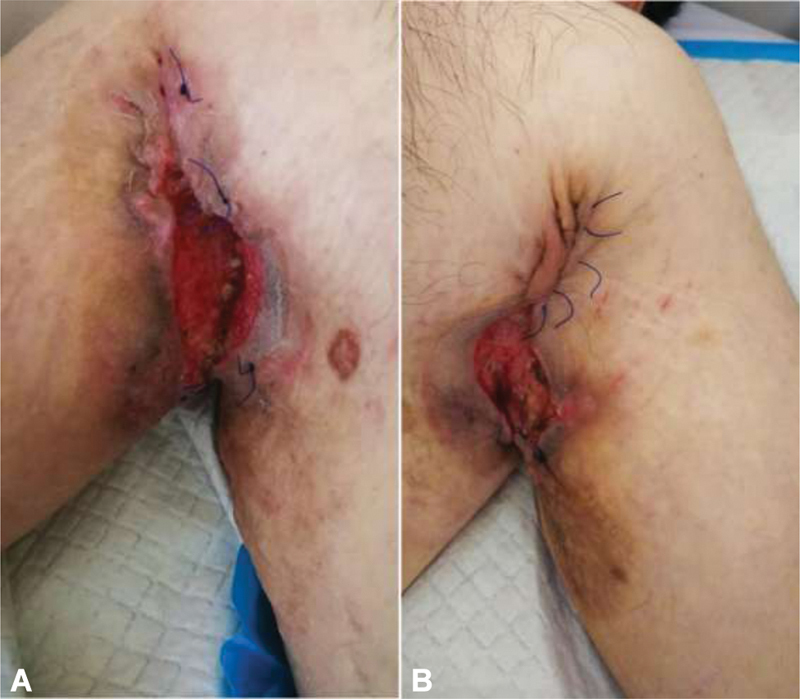
Axillary lesions excision of abscesses: ( **A** ) right axillae and ( **B** ) left axillae. Primary suture of the superior part. The inferior part kept open for drainage.

After a 6-week regimen of antibiotics, a radical excision of the lesions was done, keeping behind a wide open wound of perineum ( Fig. 2 ) and bilateral wounds of axillae ( Fig. 3 ). On day 2, a protective side colostomy was done at the level of the descending colon to divert feces.

**Fig. 4:**
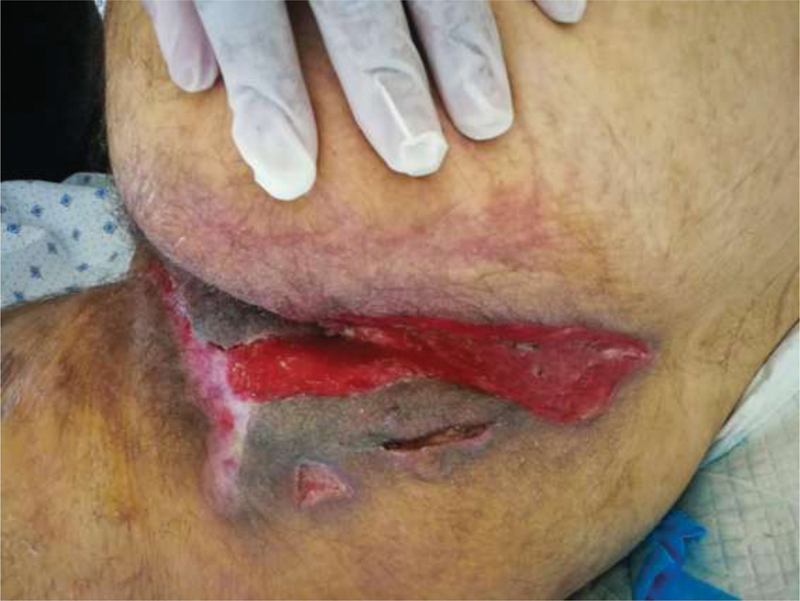
Postperineal debridement and vacuum therapy for 2 months. Granulation tissue formation noted.

Negative pressure wound therapy (Genadyne's NPWT) was initiated over the three open wounds and continued for 2 months after surgery until the granulation tissue formation was noted ( Fig. 4 ).

**Fig. 5:**
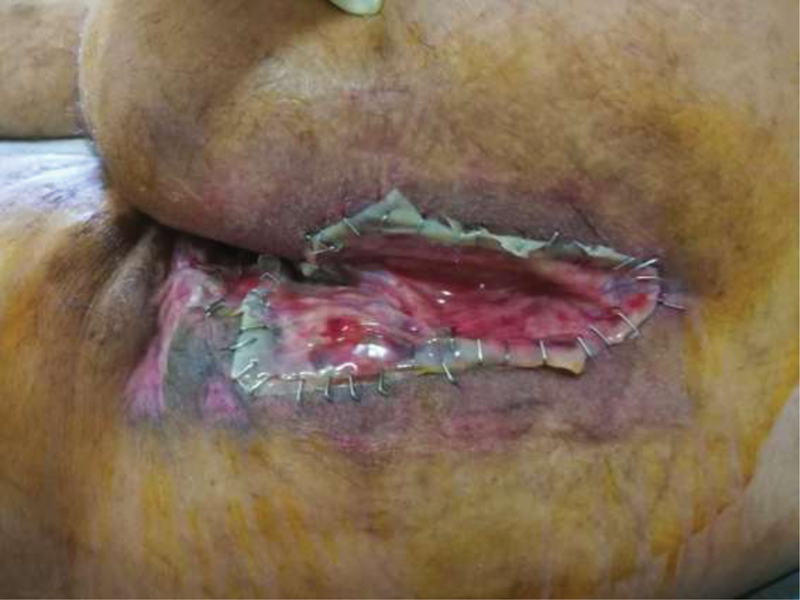
Perineal integra dermal regeneration template TM insertion and fixation.

**Fig. 6:**
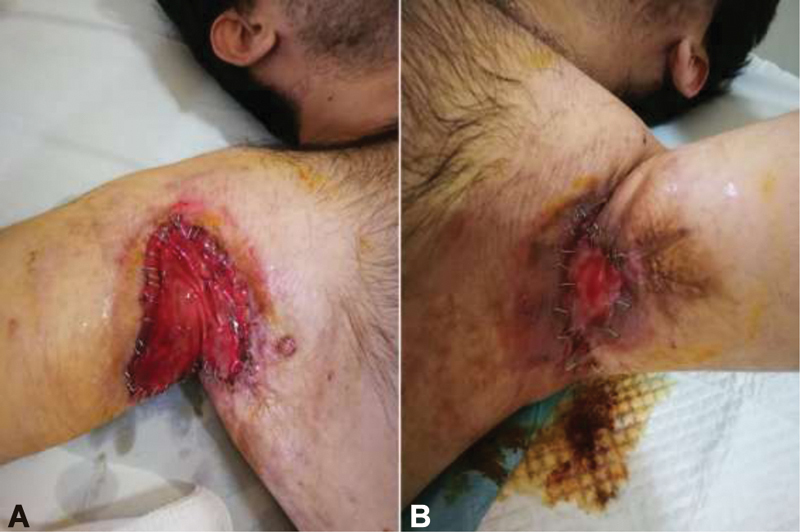
Right ( **A** ) and left ( **B** ) axillary Integra Dermal Regeneration template TM insertion and fixation.

The patient then benefited from intraoperative skin substitutes (Integra Dermal Regeneration template) placement over the open wounds and fixation with staples ( Figs. 5 and 6 ).

**Fig. 7:**
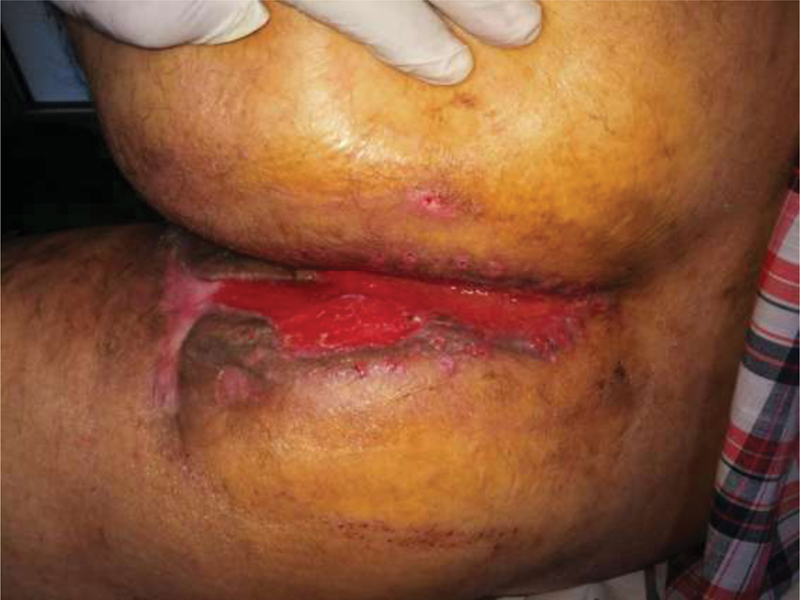
Perineal wound follow-up. In total, 3 weeks post-Integra fixation and postablation of sutures.

**Fig. 8:**
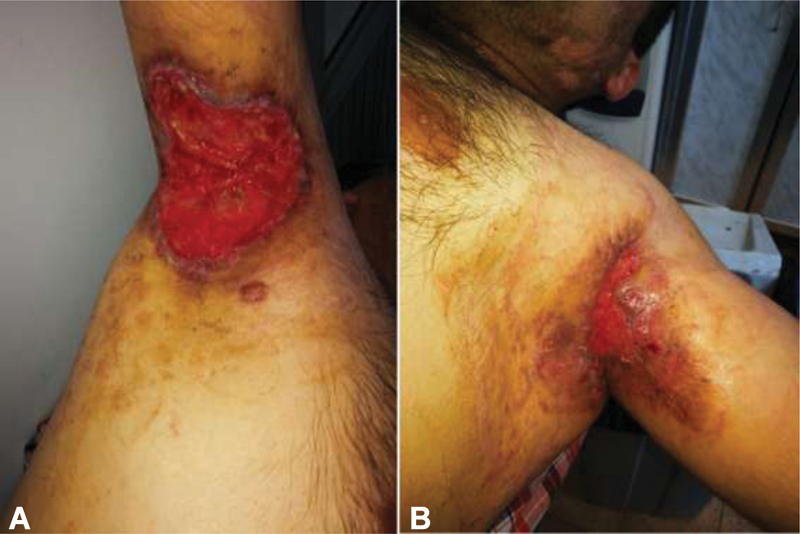
Right ( **A** ) and Left ( **B** ) axillary wounds follow-up. In total, 3 weeks post-Integra fixation and postablation of sutures.

About 3 weeks postinsertion, the follow-up demonstrated good uptake of the graft without breakdown. Surgical staples were removed and patient was discharged home with instruction of wet to dry dressing with active Leptospermum honey (MEDIHONEY Gel) ( Figs. 7 and 8 ).

**Fig. 9:**
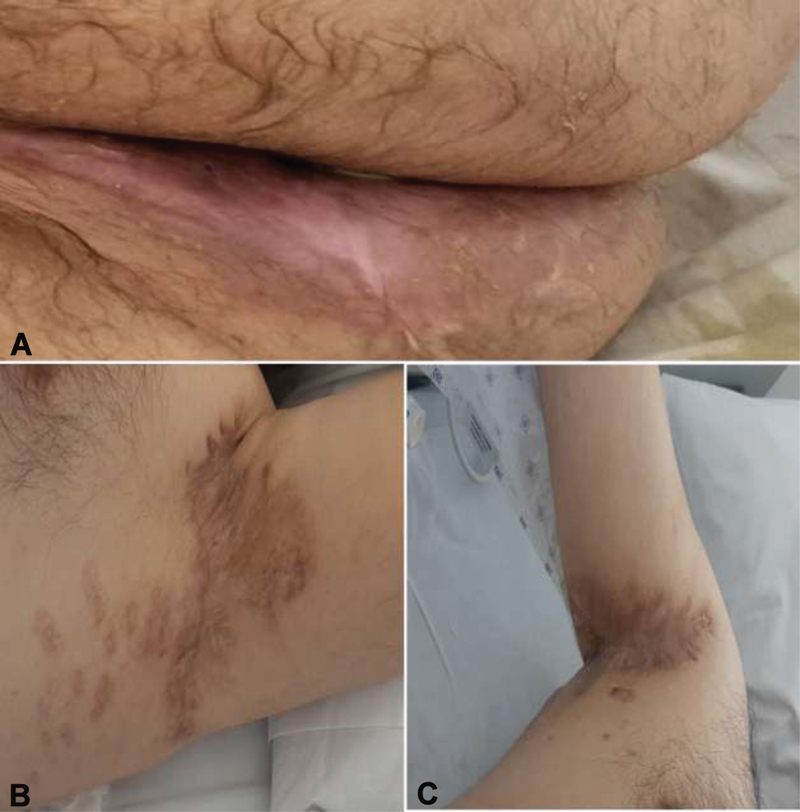
1-year follow-up post-Integra removal and without recurrences: ( **A** ) perineal, ( **B** ) left axillae, and ( **C** ) right axillae.

Moreover, 1-year follow-up showed no recurrence and preservation of patient functionality ( Fig. 9 ). Re-establishment of continuity of bowels done and patient discharged home without complications.

## Discussion

HS is a chronic inflammatory disease of the skin, with a significant recurrence and burden on the affected persons, making them infirm and anxious‎. 1 The nomenclature “hidradenitis suppurativa” is unsuitably applied, since the development of the disease involves the apocrine gland‎ 2 associated with systemic inflammation‎. 2

They are acquainted as tender abscesses that may burst repeatedly to purulent and odorous discharges‎‎. 3 They may be demarcated as an acne-like outburst, associated with excavating blackheads‎‎ 4 with endmost evolution to sinus tract development and hypertrophic scare formation‎. 3

Usually, HS are found in areas with apocrine holding skin folds such as axillae, perineum, groin, and perianal area. 1 3 It generally arises postadolescence‎ 1 (between the age of 18 and 29), with threefold incidence in females‎‎. 3 But its prevalence remains uncertain. 1

Two classification systems were developed to direct treatment and measure its efficacy: Hurley staging and the Hidradenitis Suppurativa score successively‎. 3 Due to the fact that HS is a labyrinthine and incongruous disease to date, it is so called Orphan, 5 and a variety of surgical and medical treatments were endorsed‎. 6 Nine international guidelines were elaborated recently and enclosing therapeutic manners, ranging from topical medication to interventional procedures. 1

However, treatment steadily is founded on physician clinical expertise instead of research evidence‎ 3 since these treatment modalities are vastly imperfect, and additional studies are required to inaugurate the most effective treatment guidance. 7

Forasmuch as the medical treatment takes long time to function, surgery in contrast presents instant improvement‎, 6 and it is considered the only approach with curative potential 3 mainly in the advanced cases‎, 8 even 166 years post its first description by Verneuil‎. 4

Surgical approach ranges from simple incision and drainage to local or wide excision‎. 8 Thereafter, wide excision, a reconstructive technique such as skin graft or dermal substitute application over granulation tissue should be used to cover the large defect‎. 8

**Fig. 10:**
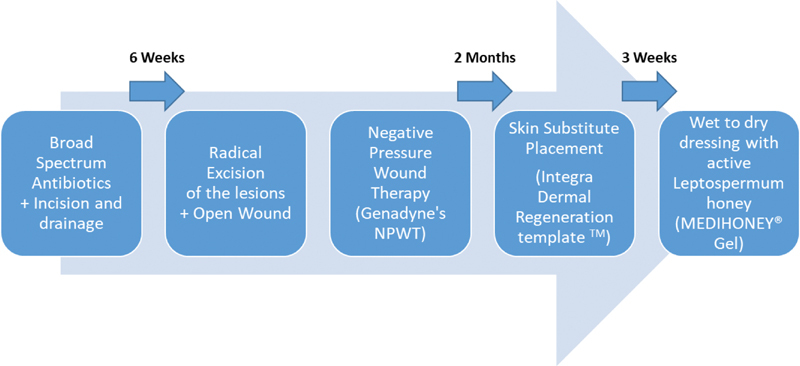
Treatment recommendation based on our own experience.

Unfortunately, there is no step-by-step guide for best practice. In the absence of described surgical technique, recurrence of this entity remains a challenge. We found it necessary to begin establishing a step-wise perspective for the treatment of such a challenging entity‎. 7 Fig. 10 summarizes our approach.

## Conclusion

This report is an attempt to describe a step-wise approach to treat HS. After one recurrence free year, we find it necessary to start broader studies and enroll our technique to end up in a well-defined recommendation for an efficient surgical and reconstructive modality for patients with Hurley Stage III HS disease in multiple simultaneous locations.
